# Evaluation of metabolism‐related molecules in rat model of autism spectrum disorders

**DOI:** 10.1113/EP092734

**Published:** 2025-09-10

**Authors:** Süeda Tunçak, Ayşen Çakır, Bülent Gören, Nevzat Kahveci

**Affiliations:** ^1^ Faculty of Medicine, Department of Physiology Bursa Uludağ University Bursa Türkiye; ^2^ Experimental Animal Breeding and Research Unit (DENHAB) Bursa Uludağ University Bursa Türkiye; ^3^ Graduate School of Health Sciences, Department of Medicine‐ Physiology Bursa Uludağ University Bursa Türkiye

**Keywords:** autism spectrum disorder, metabolism, neuropeptides, sex, valproic acid

## Abstract

Autism spectrum disorders (ASD) are neurodevelopmental pathologies. Investigating both sexes is crucial for understanding sex‐specific manifestations of ASD. This study aims to examine ASD‐like behaviours and metabolic alterations in male and female rats prenatally exposed to valproic acid (VPA). Pregnant Wistar albino rats were administered 400 mg/kg VPA or saline on embryonic day 12. Pups were subjected to various behavioural tests, including olfactory discrimination, sociability, locomotion, anxiety and exploratory behaviour assessments. On postnatal day 35, pups were sacrificed, and blood glucose levels were measured. Serum and brain leptin, orexin‐A, nesfatin‐1 and ghrelin levels were assessed by the ELISA method. VPA‐exposed pups exhibited increased latency to reach maternal bedding, reduced sociability, decreased locomotion and increased immobility in both sexes. In the elevated plus maze, VPA‐exposed females showed an increase in open‐arm entries, while males showed a reduction compared to control groups. Blood glucose levels were significantly elevated in VPA‐exposed males but not females. Significant sex‐independent changes were observed in serum and brain levels of leptin and nesfatin‐1 in the VPA groups. Brain orexin‐A and serum ghrelin levels were altered in the VPA group in a sex‐dependent manner. Prenatal VPA exposure induces ASD‐like symptoms in both sexes, with notable sex‐specific differences in behaviour and metabolic regulation. These findings highlight the importance of including both sexes in ASD research to better understand sex‐dependent characteristics of the disorder, particularly concerning metabolic dysregulation.

## Abstract (highlights)

- **What is the central question of this study?**
  Metabolic disturbances are common in autism spectrum disorders (ASD), but mechanisms linking ASD and eating disorders remain unclear: does in utero valproic acid (VPA) exposure disrupt metabolic peptides in an ASD rat model with potential sex specific effects?
- **What is the main finding and its importance?**
  Prenatal VPA exposure induces ASD‐like behaviours in male and female rats, with sex‐specific behavioural and metabolic changes. Findings suggest a shared mechanism between ASD and eating disorders, highlighting altered leptin, ghrelin and orexin levels and emphasizing the need for sex‐specific metabolic research.

## 1 INTRODUCTION

Autism spectrum disorders (ASD) are complex neurodevelopmental disorders that affect brain function. Although ASD symptoms are often seen as a spectrum, three core symptoms are necessary for diagnosis: impaired social interaction, ineffective communication and restricted/repetitive behaviours (American Psychiatric Association, 2022). Beyond these core symptoms, redox imbalance, oxidative stress and related mitochondrial disruptions have been implicated in the disorder's pathophysiology (Kaur et al., 2014). ASD is reported to have a prevalence of 2% with a male‐to‐female ratio of 4:1 (Kim et al., 2011).

Experimental models play a crucial role in understanding ASD by mimicking the environmental and genetic triggers that elicit ASD‐like behaviours, metabolic alterations and morphological changes. Valproic acid (VPA), an anticonvulsant, is associated with an increased risk of ASD in offspring when taken by the mother during the first trimester. VPA's teratogenic effects, including disruption of neural tube closure and inducing skeletal malformations (Ornoy et al., 2020), make it a widely accepted environmental trigger among ASD models (Schneider & Przewłocki, 2005). In utero exposure to VPA on embryonic day 12 (E12) results in ASD‐like symptoms in rodents, including deficits in social interaction and communication, repetitive behaviours, delays in maturation and motor development, as well as metabolic and hormonal imbalances, paralleling symptoms observed in humans (Schneider & Przewłocki, 2005). Treatments such as environmental enrichment (Schneider et al., 2006) and ketogenic diets have shown efficacy in ameliorating ASD‐like symptoms in VPA‐induced rodent models (Castro et al., 2017).

Even though there are several definitions of feeding and eating disorders (FED) in the literature, DSM‐V defines them as ‘characterized by a persistent disturbance of eating or eating‐related behaviour that results in altered consumption or absorption of food and significantly impairs physical health or psychosocial functioning’. This category includes eight disorders, with anorexia nervosa (AN), bulimia nervosa (BN), and binge‐eating disorder being the most common types (American Psychiatric Association, 2022). FED exhibits a male‐to‐female ratio of 1:5 (Carpita et al., 2022), and its pathogenesis involves a complex interplay between genetic, neurobiological and environmental factors (Culbert et al., 2015). Several hormones and neuropeptides, such as orexins, leptin, nesfatin and ghrelin, have been implicated in regulating feeding behaviour, energy metabolism and neuroendocrine function. Orexin‐A can be found in plasma, as it can cross the blood–brain barrier. It induces glucose uptake in adipocytes and increases lipogenesis (Mediavilla, 2020). Steward et al. (2019) reported that cognitive impairments in AN are related to decreased plasma levels of orexin‐A. Leptin is secreted by adipocytes and is described as a hormone that manages energy balance. Leptin levels fluctuate throughout the day, being highest at midnight and lowest early in the morning (Keleş et al., 2018). It is proposed that leptin affects orexin neural activity in order to regulate energy homeostasis (Zhu et al., 2002). Increased leptin levels have been reported in binge‐eating disorder (Turan et al., 2021). Nesfatin is an anorexigenic peptide, independent of leptin, that plays a role in the regulation of feeding (Maejima et al., 2009). Nesfatin‐1 is associated with diabetes and obesity, as well as some psychiatric disorders and neurogenic diseases (Dai et al., 2013). Experimental studies in rodents have shown that nesfatin‐1 exerts inhibitory effects on gastroduodenal motility and gastric emptying. Furthermore, central administration of nesfatin‐1 at doses effective in reducing food intake also suppresses vagally mediated gastric acid secretion. These findings suggest that nesfatin‐1 plays a dual regulatory role in both gastrointestinal motility and gastric secretory function, potentially contributing to its anorexigenic effects (Xia et al., 2012). Increased anxiety in people with AN is reported to be associated with increased plasma nesfatin‐1 levels (Weibert et al., 2019). Ghrelin is an orexigenic hormone that promotes food intake, and its altered plasma levels have been reported in FED (Atalayer et al., 2013).

Despite DSM‐V not listing unusual eating habits or problems with food intake as main symptoms of ASD, these disruptions are reported as early signs of the disorder. Approximately 90% of children with ASD exhibit feeding problems (Kodak & Piazza, 2008). The prevalence of ASD in women is estimated at 1%, but this rate rises to 20–35% among women with AN (Westwood & Tchanturia, 2017). Recently, it has been suggested that problems related to food intake, including AN, can be female‐specific symptoms of ASD and that they are often overlooked due to male biases in ASD. Restricted interests and repetitive behaviours, which are main symptoms of ASD, are suggested to manifest as problems with food intake in females. There are also reported similarities in the insistence on rituals and stereotypical behaviours between people with AN and ASD (Carpita et al., 2022).

Physiological similarities between ASD and FED, such as metabolic disturbances, further support this link. Increased plasma (Ashwood et al., 2008) and anterior cingulate gyrus (L. Chen et al., 2024) levels of leptin and decreased plasma ghrelin (Al‐Zaid et al., 2014) levels have been reported in individuals with ASD. In a recent study, orexin receptor antagonist application has been shown to improve ASD‐like symptoms in rats, suggesting a possible role of the orexinergic system in ASD pathogenesis (Piri et al., 2024).

As mentioned above, the similarities in both physiological and behavioural symptoms between ASD and FED, along with the frequent reporting of metabolic disturbances in ASD, highlight the importance of investigating the relationship between these conditions. Additionally, the improvement of ASD symptoms with dietary adjustments underscores the need to explore the role of key hormones and peptides involved in metabolism and eating behaviours.

In this study, ASD was modelled by In utero VPA exposure in rats. Both female and male offspring were assessed for ASD‐like symptoms. The levels of key peptides involved in metabolic regulation were analysed in serum and brain tissues.

## 2 METHODS

### 2.1 Ethical approval

The study was approved by the Bursa Uludağ University Local Ethics Committee on Animal Research under decision number 2024‐01/08. The authors confirm that the study strictly adheres to *Experimental Physiology*’s policies regarding animal experiments.

### 2.2 Experimental animals and timeline

All animals were housed under standard laboratory conditions at the Bursa Uludağ University Experimental Animal Breeding and Research Unit. The environment was maintained on a 12‐h light–dark cycle, with controlled temperature (22–25°C) and humidity (40–60%), and animals had access to food and water ad libitum. All experiments were performed during the light phase.

Wistar Albino females and males were mated at a 2:1 ratio overnight. Pregnancy was confirmed by the presence of spermatozoa in vaginal smear samples, which was designated as E0. Pregnant rats were randomly divided into two groups: the model group received 400 mg/kg VPA intraperitoneal (i.p.) (BD41139, BLDpharm, Shanghai, China) (*n*_Mother_ = 4), and the control group received saline (S, 1 mL/kg) (*n*_Mother_ = 4) i.p. on E12.5. The dosage of 400 mg/kg VPA was selected based on previous studies that demonstrated the induction of ASD‐like symptoms with reduced toxicity and reabsorption rates (Cezar et al., 2018; Morales‐Navas et al., 2024). The day of birth was designated as postnatal day 0 (P0). On P21, pups were weaned and caged with siblings, with an average of four animals per cage. Both female and male pups, with a maximum of two pups per sex per litter, were randomly selected and used for further experiments. Pups were divided into four groups: SAL‐F (females that were prenatally exposed to saline; *n* = 8), SAL‐M (males that were prenatally exposed to saline; *n* = 8), VPA‐F (females that were prenatally exposed to VPA; *n* = 8) and VPA‐M (males that were prenatally exposed to VPA; *n* = 8). None of the animals died before the experiments were concluded.

**FIGURE 1:**
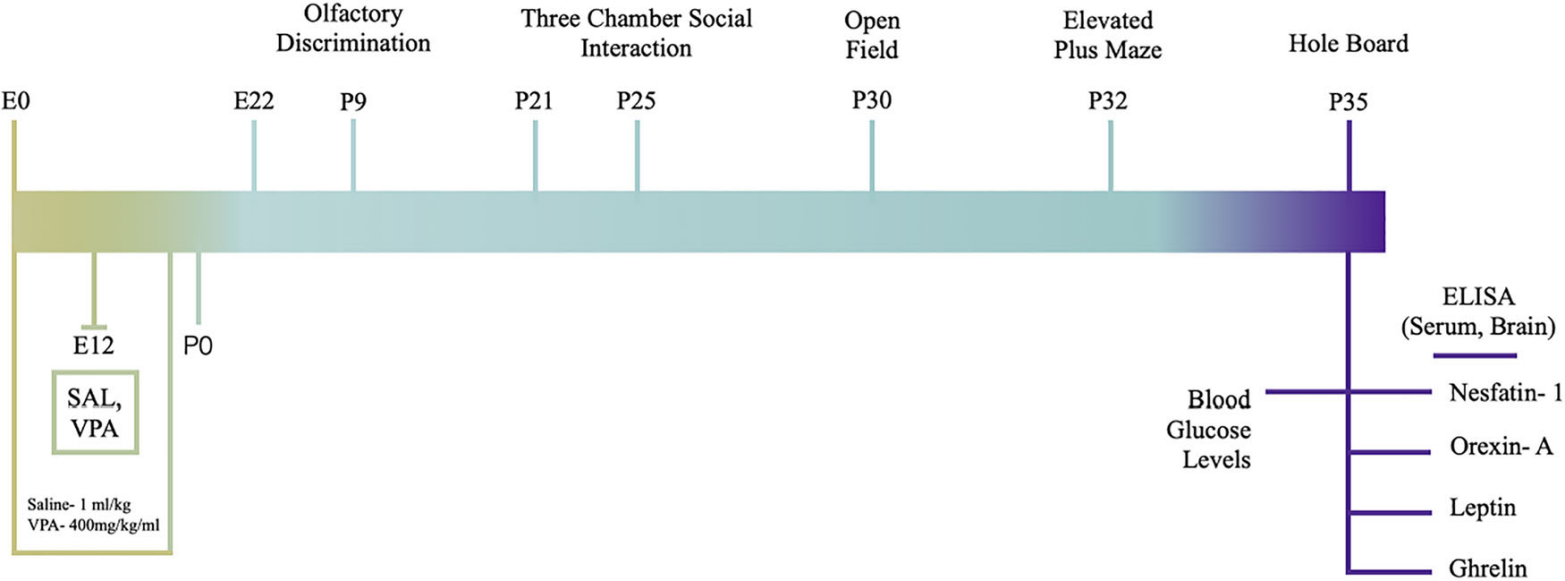
Experimental timeline. E, embryonic day; P, postnatal day.

To assess the face validity of the model, pups were tested for olfactory discrimination (OD) on P9 and sociability on P25. Afterwards, locomotion was assessed on P30, anxiety was tested on P32 and exploratory behaviour was examined on P35. Pups were decapitated on P35 following a 16‐h fasting period. Blood glucose levels were measured immediately. Nesfatin‐1, orexin‐A, leptin and ghrelin levels were analysed in both serum and brain homogenates using commercial ELISA kit protocols (Figure 1). Oestrous cycle assessment was not necessary, as female rats do not begin cycling until P38–39 (Ajayi & Akhigbe, 2020). Analysis was conducted blind to the experimental conditions.

### 2.3 Behavioural analyses

All behavioural analyses were conducted on the same eight animals per group.

#### 2.3.1 Olfactory discrimination

Pups were separated from their mothers for 5 min prior to testing on P9. The test was performed in a transparent plastic chamber (20 × 8 × 8 cm^3^) divided into five invisible areas, each with dimensions 4 × 8 cm. Three areas in the middle were left empty, while one side area had bedding from the mother's cage and the other side area had clean bedding (Schneider & Przewłocki, 2005). The subject pup was placed in the centre of the chamber, and its activity was recorded for 3 min by a fixed camera. Latency to reach any bedding was measured. Pups were returned to their mothers immediately once they reached any bedding. The chamber was cleaned with 70% alcohol after each test to avoid any odour marks.

#### 2.3.2 Sociability

The three‐chambered social performance test was used to assess the sociability of same‐sex rats on P25 (Kaidanovich‐Beilin et al., 2010). The apparatus consisted of wire‐meshed containers and two larger side chambers (25 × 25 × 25 cm^3^) with a smaller central chamber (10 × 25 × 25 cm^3^), all accessible through openings. The wire‐meshed containers were wide enough to allow pups to move easily inside. Visual, auditory, olfactory and tactile interactions were possible between the inside and outside of the containers. Subject animals were habituated to the chambers for 5 min prior to testing. A same‐sex, same‐strain stranger rat in a wire‐meshed container was placed into one of the side chambers, while the other chamber was left empty. The subject rat was then placed in the central chamber. Its activity was recorded for 5 min by a fixed camera. The time spent in each chamber was analysed from the recordings. The chambers and containers were cleaned with 70% alcohol after testing to avoid any odour marks.

#### 2.3.3 Open field

Locomotor activity of each rat was tested using an open field (OF) test on P30. Measurements were conducted in a 40 × 40 × 40 cm^3^ black‐floored Plexiglas box for 10 min. Their movements were recorded and analysed using EthoVision XT15 software (Noldus, Amsterdam, Netherlands). Parameters to evaluate locomotor activity included total walking distance and duration of immobility. An animal was considered immobile when its mobility was below 4% in a given location, and grooming was not included. The box was cleaned after each test with 70% alcohol to avoid any odour marks. The room was dimly illuminated (8–10 lux).

#### 2.3.4 Elevated plus maze

Anxiety was measured using the elevated plus maze (EPM) on P31. The apparatus consisted of two open arms (50 × 10 cm^2^) and two closed arms (50 × 10 × 40 cm^3^) with a merged central area. The subject rat was placed in the central area, facing an open arm, and its activity was recorded by a fixed camera for 5 min. The frequency of entering open/closed arms was measured through the recordings, which were transformed into numerical data (EthoVision XT15 software). The apparatus was cleaned with 70% alcohol between tests to avoid any odour marks.

#### 2.3.5 Hole‐board test

Exploratory behaviour was evaluated using the hole‐board test (HBT) on P35. The apparatus consisted of a Plexiglas box (50 × 50 × 50 cm^3^) with a black platform containing 16 evenly spaced circular holes (2 cm in diameter). The subject rat was placed in the centre of the box, and its activity was recorded by a fixed camera for 5 min (Kumar et al., 2015). The frequency of head‐dipping was measured through the recordings. The apparatus was cleaned with 70% alcohol after each test to avoid any odour marks.

### 2.4 Molecular analyses

On completion of experiments, rats were sacrificed under sevoflurane (3%) anaesthesia by decapitation on P35 after 16 h of fasting. Blood was collected post‐decapitation at 10.00 h. Blood glucose levels were measured using an AccuCheck glucometer (Roche, Basel, Switzerland). Blood samples were centrifuged for 20 min at 1030 RCF; serum was collected and stored at −80°C until use (*n* = 8 per group). Whole brains were collected and homogenized following the kit protocols (*n* = 7 per group). Nesfatin‐1, orexin‐A, leptin and ghrelin levels were analysed spectrophotometrically from serum and brain homogenates, following commercial kit protocols based on the ELISA principle (BT‐LAB, Shanghai Korain Biotech Co., Ltd, Shanghai, China). The steps of the ELISA procedure were as follows: first, all reagents, samples and standards were prepared. Then, the sample and ELISA reagent were added into each well and incubated for 1 h at 37°C. After incubation, the plate was washed 5 times. Substrate solutions A and B were added, followed by another 10‐min incubation at 37°C. Finally, the stop solution was added to allow colour development, and the optical density (OD) value was measured at 450 nm within 10 min.

### 2.5 Statistical analyses

In order to evaluate possible interaction between sex and VPA exposure, a two‐way ANOVA was performed after confirming normality and equal variance assumptions. If a significant difference was detected (*P*_ANOVA_ < 0.05), a *post hoc* test was conducted using the Holm–Šidák test. Student's *t*‐test was performed to compare litter size and time spent in empty and social chambers in the sociability test. All statistical analyses were performed on SigmaPlot (Systat Software, Inc., San Jose, CA, USA), and significance was accepted as *P *< 0.05. Group means with standard deviation are presented.

## 3 RESULTS

### 3.1 Demographic data

None of the control animals (*n*_SAL‐F_ = 8, *n*_SAL‐M_ = 8) displayed any physical malformations. However, among the VPA animals (*n*_VPA‐F_ = 8, *n*_VPA‐M_ = 8), 6 out of 16 displayed malformations in the form of bent tails. Student's *t*‐test showed no significant difference in litter size between groups (Table 1).

**TABLE 1:** Distribution of pups per litter per group.

Group	Mother	Number of pups	
		Female	Male
SAL	S1	4	5
	S2	6	7
	S3	7	3
	S4	5	6
VPA	V1	6	6
	V2	4	7
	V3	5	3
	V4	3	6

Abbreviations: SAL, saline; VPA, valproic acid.

**FIGURE 2:**
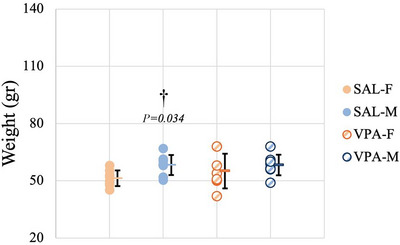
Body weight on P35. Data are presented as means ± SD (*n*_SAL‐F_ = 8, *n*_SAL‐M_ = 8, *n*_VPA‐F_ = 8, *n*_VPA‐M_ = 8). † indicates comparison between females and males. Orange columns represent females, blue columns represent males, filled circles represent SAL groups, and hatched circles represent VPA groups.

All animals were weighed before sacrifice on P35. Data for body weight passed both the normality and equal variance tests. There was no significant interaction between sex and VPA exposure after two‐way ANOVA (*F*(1.31) = 0.778, *P *= 0.385). SAL‐F weighed significantly less compared to SAL‐M (*P *= 0.034) (Figure 2).

### 3.2 Olfactory discrimination

**FIGURE 3:**
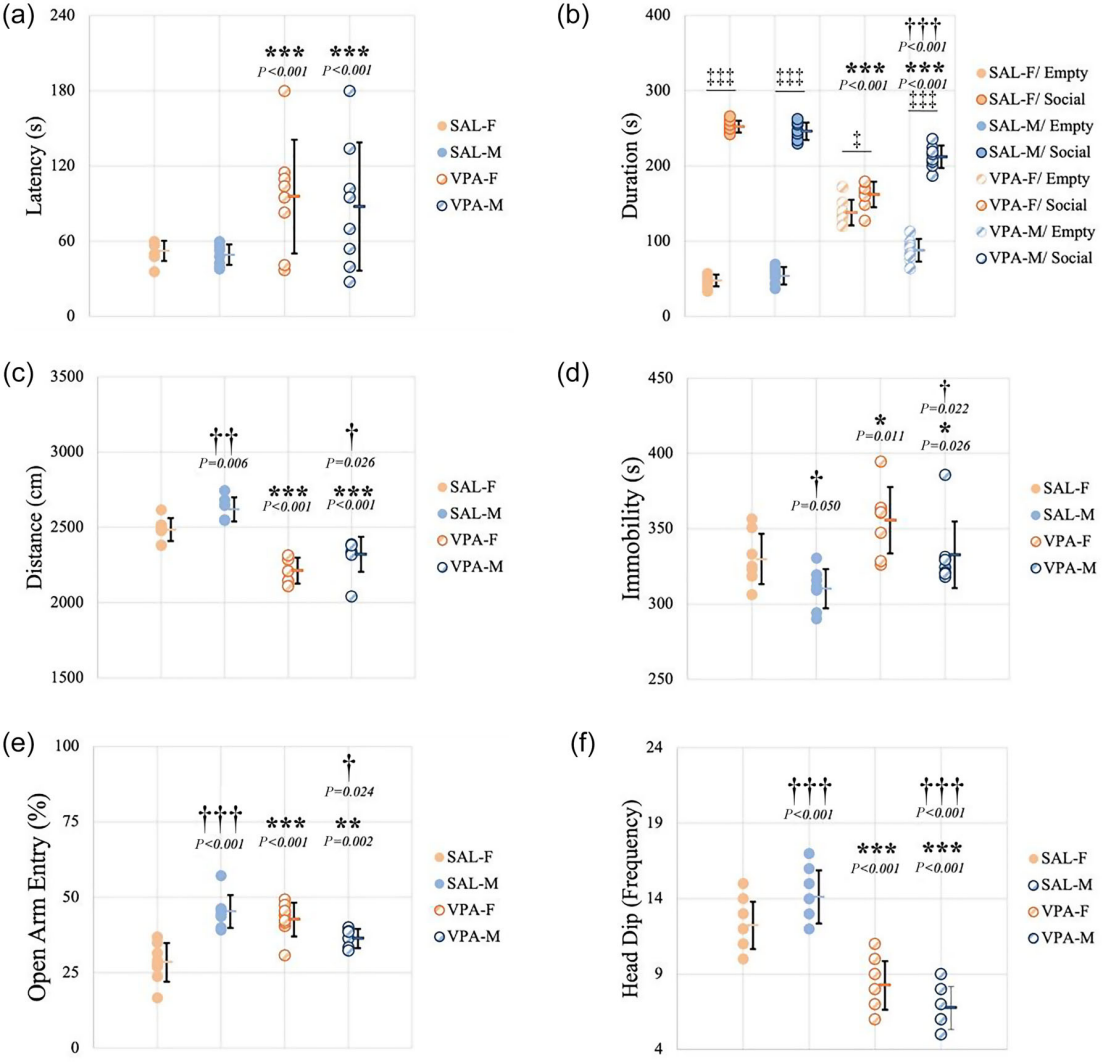
Behavioural analyses. (a) Olfactory discrimination – latency to reach mother. (b) Sociability – time spent in empty and social chambers. (c) Locomotor activity – distance moved in open field. (d) Locomotor activity – duration of immobility in open field. (e) Elevated plus maze – ratio of entry to open arms. (f) Hole‐board test – frequency of head dipping. Data are presented as means ± SD (*n*_SAL‐F_ = 8, *n*_SAL‐M_ = 8, *n*_VPA‐F_ = 8, *n*_VPA‐M_ = 8). * indicates comparison between SAL and VPA groups; † indicates comparison between females and males; ‡ indicates comparison between empty and social chambers. Orange columns represent females; blue columns represent males; filled circles represent SAL groups; and hatched circles represent VPA groups.

Animals were tested for OD on P9. Data for OD passed both normality and equal variance tests. There was no significant interaction between sex and VPA exposure after two‐way ANOVA (*F*(1.31) = 0.515, *P *= 0.479). *Post hoc* analyses revealed that the VPA‐F group displayed significantly increased latency to reach maternal bedding compared to SAL‐F (*P *< 0.001). VPA‐M also showed increased latency to reach maternal bedding compared to SAL‐M (*P *< 0.001). There were no significant differences between sexes within saline or VPA treatments (Figure 3a).

### 3.3 Sociability

Pups were tested for sociability on P25. Preference between empty and social chambers was first analysed within groups. Student's *t*‐test showed that all groups had an increased tendency to spend time in the social chamber (*P*_SAL‐F_ < 0.001; *P*_SAL‐M_ < 0.001; *P*_VPA‐F_ = 0.012; *P*_VPA‐M_ < 0.001). All groups were then compared with two‐way ANOVA, and there was a significant interaction between sex and VPA (*F*(1.31) = 43.175, *P *< 0.001). *Post hoc* analyses then revealed that both VPA‐F and VPA‐M groups spent significantly less time in the chamber containing the stranger rat compared to SAL‐F and SAL‐M, respectively (*P *< 0.001 and *P *< 0.001). VPA‐F also spent significantly less time in the chamber containing the stranger rat compared to VPA‐M (*P *< 0.001); however, no sex differences was observed in the control group (Figure 3b).

### 3.4 Open field

**FIGURE 4:**
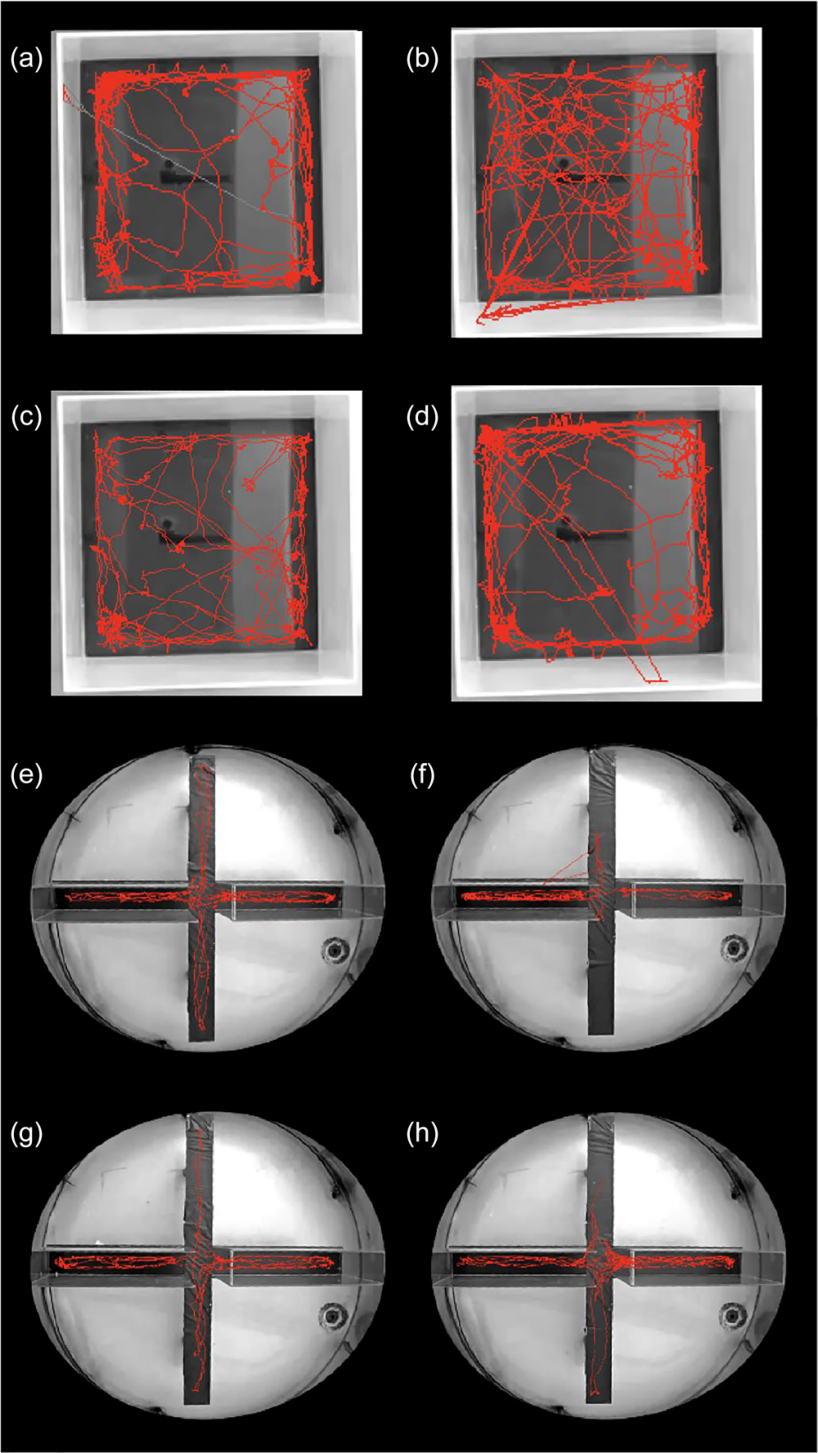
Representative tracking images for open field: (a) SAL‐F, (b) SAL‐M, (c) VPA‐F, (d) VPA‐M; and for the elevated plus maze: (e) SAL‐F, (f) SAL‐M, (g) VPA‐F, (h) VPA‐M.

Locomotor activity was measured on P30. Distance moved and duration of immobility were analysed. There was no significant interaction between sex and VPA exposure for distance moved after two‐way ANOVA (*F*(1.31) = 0.189, *P *= 0.667). According to *post hoc* analyses, both VPA‐F and VPA‐M travelled significantly less compared to SAL‐F and SAL‐M, respectively (*P *< 0.001 and *P *< 0.001). There were also significant differences between sexes within treatment. Both SAL‐F and VPA‐F travelled significantly less compared to SAL‐M and VPA‐M, respectively (*P *= 0.006 and *P *= 0.026) (Figure 3c). Two‐way ANOVA revealed that there was no significant interaction between sex and VPA exposure for duration of immobility (*F*(1.31) = 0.0734, *P *= 0.788). VPA‐F and VPA‐M spent significantly more time immobile compared to SAL‐F and SAL‐M (*P *= 0.011 and *P *= 0.026). *Post hoc* analyses also revealed that SAL‐F and VPA‐F were more immobile compared to SAL‐M and VPA‐M, respectively (*P *= 0.05 and *P *= 0.022) (Figure 3d). Representative movement‐tracking traces can be found in Figure 4a–d.

### 3.5 Elevated plus maze

Anxiety was measured on P32. The frequency of entering open and closed arms was analysed. There was a significant interaction between sex and VPA according to two‐way ANOVA (*F*(1.31) = 38.323, *P *< 0.001). *Post hoc* analyses then revealed that VPA‐M showed a significantly lower ratio of open arm entry compared to SAL‐M (*P *= 0.002). On the contrary, VPA‐F showed a significantly higher ratio of open arm entry compared to SAL‐F (*P *< 0.001) and VPA‐M (*P *= 0.024). SAL‐M showed a significantly higher ratio compared to SAL‐F (*P *< 0.001) (Figure 3e). Representative movement‐tracking traces can be found in Figure 4e–h.

### 3.6 Hole‐board test

Exploratory activity was measured on P35. The frequency of head‐dipping was analysed. After two‐way ANOVA, there was a significant interaction between sex and VPA (*F*(1.31) = 34.300, *P *< 0.001). VPA‐F displayed significantly decreased frequency of head‐dipping compared to SAL‐F (*P *< 0.001). However, the group had a higher frequency compared to VPA‐M (*P *< 0.001). VPA‐M displayed significantly decreased frequency of head‐dipping compared to SAL‐M (*P *< 0.001). SAL‐F also showed decreased frequency of head‐dipping compared to SAL‐M (*P *< 0.001) (Figure 3f).

### 3.7 Blood glucose

**FIGURE 5:**
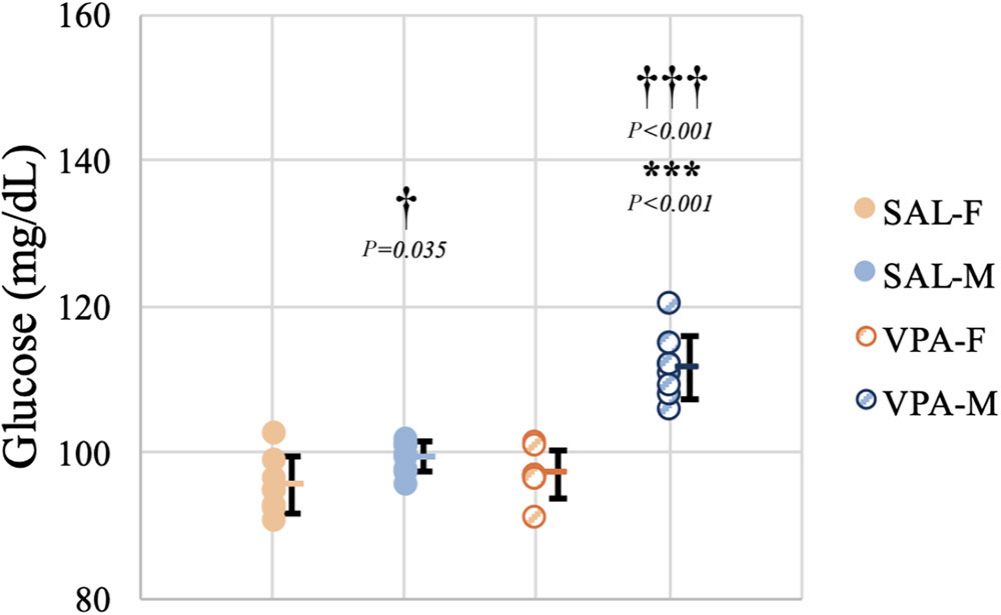
Blood glucose levels. Data are presented as means ± SD (*n*_SAL‐F_ = 8, *n*_SAL‐M_ = 8, *n*_VPA‐F_ = 8, *n*_VPA‐M_ = 8). * indicates comparison between SAL and VPA groups and † indicates comparison between females and males. Orange columns represent females, blue columns represent males, filled circles represent SAL groups, and hatched circles represent VPA groups.

Blood glucose levels were measured on P35 after 16 h of fasting. There was a significant interaction between sex and VPA in blood glucose levels (*F*(1.31) = 20.145, *P *< 0.001). VPA‐F and SAL‐F showed no significant difference (*P *= 0.383). However, VPA‐M showed significantly increased levels of blood glucose compared to SAL‐M (*P *< 0.001) and VPA‐F (*P *< 0.001). SAL‐M also showed higher blood glucose levels compared to SAL‐F (*P *= 0.035) (Figure 5).

### 3.8 Nesfatin‐1 levels

**FIGURE 6:**
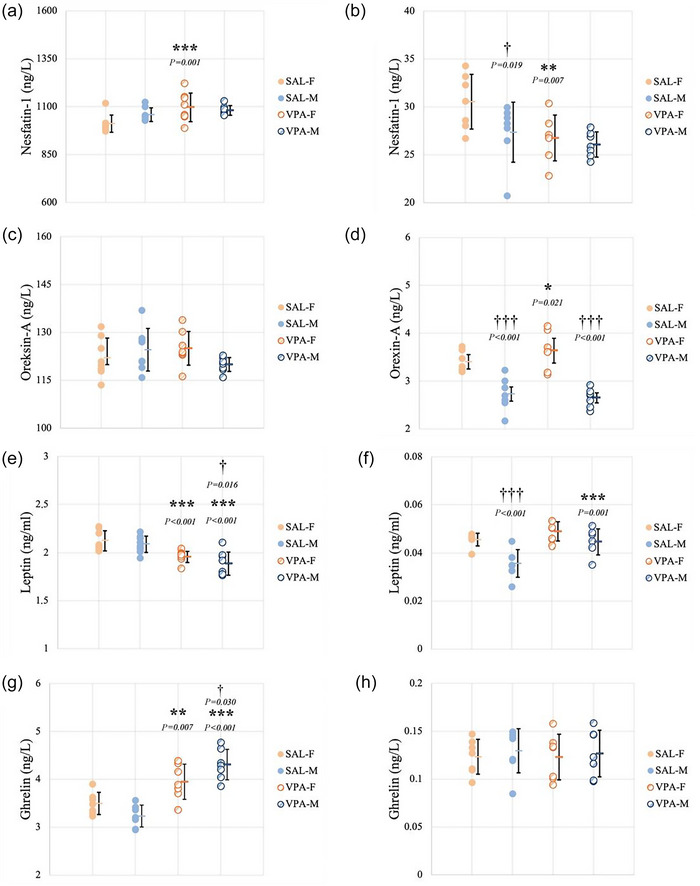
Molecular analyses. ELISA results for levels of (a) serum nesfatin‐1, (b) brain nesfatin‐1, (c) serum orexin‐A, (d) brain orexin‐A, (e) serum leptin, (f) brain leptin, (g) serum ghrelin, (h) brain ghrelin. Data are presented as means ± SD (serum: *n*_SAL‐F_ = 8, *n*_SAL‐M_ = 8, *n*_VPA‐F_ = 8, *n*_VPA‐M_ = 8; brain: *n*_SAL‐F_ = 7, *n*_SAL‐M_ = 7, *n*_VPA‐F_ = 7, *n*_VPA‐M_ = 7). * indicates comparison between SAL and VPA groups and † indicates comparison between females and males. Orange columns represent females, blue columns represent males, filled circles represent SAL groups, and hatched circles represent VPA groups.

Nesfatin‐1 was measured in both serum and whole brain samples. There was no significant interaction between sex and VPA exposure for serum after two‐way ANOVA (*F*(1.31) = 3.453, *P *= 0.074). *Post hoc* analyses revealed that VPA‐F had higher levels of serum nesfatin‐1 compared to SAL‐F (*P *= 0.001) (Figure 6a). Nesfatin‐1 levels in brain homogenates did not display any significant interaction between sex and VPA exposure (*F*(1.27) = 1.992, *P *= 0.171). VPA‐F had significantly lower levels of nesfatin‐1 in the brain compared to SAL‐F (*P *= 0.007). There was also a significant difference between sexes in the saline group. SAL‐M showed lower nesfatin‐1 levels in brain homogenates compared to SAL‐F (*P *= 0.019) (Figure 6b).

### 3.9 Orexin‐A levels

Orexin‐A was measured in both serum and whole brain samples. Two‐way ANOVA found no significant interaction between sex and VPA exposure for serum (*F*(1.31) = 4.067, *P *= 0.053). There was no significant difference in serum orexin‐A for any group (Figure 6c). Orexin‐A levels in brain homogenates displayed a significant interaction between sex and VPA exposure (*F*(1.27) = 5.535, *P *= 0.027). The VPA‐F group showed significantly increased brain orexin‐A levels compared to both the SAL‐F (*P *= 0.021) and VPA‐M (*P *< 0.001) groups. VPA‐M and SAL‐M groups did not show any differences; however, the SAL‐M group showed lower levels of brain orexin‐A compared to the SAL‐F group (*P *< 0.001) (Figure 6d).

### 3.10 Leptin levels

Leptin was measured in both serum and whole brain samples. There was no significant interaction between sex and VPA exposure for serum after two‐way ANOVA (*F*(1.31) = 0.896, *P *= 0.352). Both the VPA‐F and VPA‐M groups showed significantly decreased levels of serum leptin compared to SAL‐F and SAL‐M, respectively (*P *< 0.001; *P *< 0.001). *Post hoc* analyses also revealed a significant difference between sexes within VPA groups. VPA‐M showed decreased levels of serum leptin compared to VPA‐F (*P *= 0.016) (Figure 6e). Leptin levels in brain homogenates did not display any significant interaction between sex and VPA exposure (*F*(1.27) = 2.402, *P *= 0.134). The VPA‐M group had significantly increased levels of leptin in the brain compared to SAL‐M (*P *= 0.001). There was also a significant difference between sexes within saline groups. SAL‐M showed lower leptin levels in brain homogenates compared to SAL‐F (*P *< 0.001) (Figure 6f).

### 3.11 Ghrelin levels

Ghrelin was measured in both serum and whole brain samples. Two‐way ANOVA revealed a significant interaction between sex and VPA exposure for serum (*F*(1.27) = 8.077, *P *= 0.009). According to *post hoc* analyses, the VPA‐F group showed an increased level of serum ghrelin compared to the SAL‐F group (*P *= 0.007). The VPA‐M group also showed increased serum ghrelin levels compared to both the VPA‐F (*P *= 0.030) and SAL‐M groups (*P *< 0.001) (Figure 6g). Ghrelin levels for brain homogenates did not display any significant interaction between sex and VPA exposure (F(1.27) = 0.0509, *P *= 0.823). There was no significant difference in serum ghrelin for any group (Figure 6h).

## 4 DISCUSSION

The present study provides novel insights into the behavioural changes and alterations in serum and brain levels of leptin, orexin‐A, nesfatin‐1 and ghrelin in female and male rats following prenatal VPA exposure. VPA is a reported environmental risk factor for ASD and is widely used to model ASD experimentally (Melancia et al., 2018). In utero VPA exposure induces social and communicative deficits, repetitive behaviours and impairments in physical and motor development in rodents (Schneider & Przewłocki, 2005). The prenatal VPA exposure model offers a useful framework for studying sex differences in ASD‐related behaviours, particularly given reports that VPA‐exposed children show a male‐to‐female ASD ratio closer to 1:1 (Rasalam et al., 2005), compared to the commonly cited 4:1 ratio in idiopathic ASD. However, in rodent models, the sex‐specific expression of ASD‐like traits remains poorly understood and requires systematic investigation using adequately powered designs. Although our study included equal numbers of male and female offspring, the small sample size and limited number of litters prevent us from drawing conclusions regarding sex‐specific prevalence or distribution of traits. Rather than addressing prevalence, our aim was to ensure both sexes were represented to allow exploratory observations of potential sex differences in behavioural responses to VPA. Recent human meta‐analyses, meanwhile, suggest the male‐to‐female ASD ratio may be closer to 3.25:1 (Loomes et al., 2017). While the higher prevalence of ASD in males has often been attributed to sex‐specific genetic factors like single nucleotide polymorphisms, single nucleotide variants, copy number variants and microdeletions (Sato et al., 2012; Tropeano et al., 2013, 2016), recent perspectives propose alternative explanations, suggesting that ASD risk genes might not be inherently sex‐biased but could instead interact with sexually dimorphic biological systems, including hormonal or immune pathways (McCarthy & Wright, 2017; Werling et al., 2016). Another intriguing possibility is that female embryos may be more vulnerable to severe genetic perturbations, potentially leading to selective attrition, though this hypothesis requires further validation (McCarthy & Wright, 2017). Females with ASD may be underdiagnosed or misdiagnosed due to subtler symptoms, better masking, or the presence of symptoms not included in conventional diagnostic criteria (Ferri et al., 2018). ASD research also does not include females as much as males due to the reported male‐to‐female ratio, which risks reducing the power of statistical analyses. These emerging concepts underscore the importance of including both sexes in ASD research and utilizing animal models to investigate how developmental manipulation of sex hormones and related molecular pathways might contribute to the observed sex differences.

Disrupted communication is one of the core symptoms of ASD (American Psychiatric Association, 2022). In rodents, communication deficits can be assessed using olfactory discrimination tests, as olfaction plays a key role in communication. According to previous studies, prenatal VPA exposure impairs olfactory discrimination, as reflected by the inability to differentiate between maternal and clean bedding (Schneider & Przewłocki, 2005). In line with these findings, our study revealed that VPA‐exposed pups exhibited reduced latency to reach maternal bedding, regardless of sex.

Deficiencies in social interaction are another core symptom that should be observed in ASD models. Healthy rodents exhibit a preference for spending time with novel animals. However, this tendency becomes avoidance in rats that were prenatally exposed to VPA (Mabunga et al., 2015). Our findings demonstrated that VPA‐exposed animals avoided novel conspecifics, consistent with the literature. Additionally, this avoidance occurred in a sex‐dependent manner, with females being more avoidant compared to males. Melancia et al. (2018) reported that both females and males show differences in social interaction during adolescence, but these differences persist into adulthood only in males.

Reports on locomotor activity in VPA‐induced ASD rodent models have been inconsistent. Schneider & Przewłocki (2005) observed increased immobility with no significant changes in the distance travelled, while Sandhya et al. (2012) reported increased locomotion. Our results showed that VPA‐exposed animals exhibited reduced movement and increased immobility compared to controls, independent of sex. In contrast to the findings of Degroote et al. (2014), who reported no sex differences in locomotor activity during open‐field testing, our results demonstrated significantly reduced activity in female rats compared to males across both VPA‐exposed and control groups. This sexual dimorphism in baseline locomotor activity is supported by existing literature, though with notable variability between studies. Kim et al. (2013) observed male‐specific hyperlocomotion, while Raza et al. (2015) documented increased locomotor activity in both sexes, with greater magnitude in males (Kim et al., 2013; Raza et al., 2015). The observed inconsistencies across studies may reflect methodological heterogeneity, including variations in rodent strains, experimental protocols and environmental conditions (Chaliha et al., 2020).

Increased anxiety has been reported in both ASD and FED. Among different methods to measure anxiety‐like behaviour in rodents, the EPM is a widely used method. Entry into open arms indicates less anxiety and more exploratory behaviour. Previous studies have reported that VPA‐exposed animals tend to avoid open arms and spend more time in the closed arms (DeCoteau & Fox, 2022). Our findings revealed decreased open‐arm entries in male VPA‐exposed rats, consistent with previous studies using only male rats. However, female VPA‐exposed rats showed increased open‐arm entries compared to control females, while control females exhibited fewer open‐arm entries than control males. This sex‐dependent difference may indicate distinct patterns of anxiety alteration in female rats.

Exploratory behaviour was assessed using the hole‐board test. Sandhya et al. and Kumar et al. showed a decreased number of head dips in VPA‐exposed animals (Kumar et al., 2015; Sandhya et al., 2012). Melancia et al. (2018), on the other hand, suggested that the hole‐board test can be used to assess repetitive behaviour and reported an increased number of head dips in VPA‐exposed animals. Our results are consistent with those of Kumar et al. (2015), showing that males were more vulnerable to impaired exploratory behaviour compared to females. Reduced exploration in the VPA group is suggested to be due to abnormal fear conditioning, which decreases locomotion and exploration (Sandhya et al., 2012).

Previous research has demonstrated that prenatal VPA exposure (600 mg/kg) significantly decreased open‐arm time in male offspring in the EPM, while no significant effect was observed in females. Conversely, prenatal VPA increased rearing frequency exclusively in females. In contrast, postnatal VPA administration (400 mg/kg) elicited anxiety‐like behaviours predominantly in females, as evidenced by reduced time spent in the central zone of the open field test (OFT), whereas males exhibited heightened grooming behaviour. These findings indicate a sexually dimorphic response to VPA exposure: prenatal VPA preferentially induces anxiety‐related behavioural deficits in males, while postnatal VPA primarily impacts females (Shafaghi et al., 2022). In a study by Thornton et al. (2021), it was reported that prenatal VPA exposure induced sex‐specific behavioural differences in adolescent rats. Specifically, males showed reduced social responsiveness in the three‐chamber and olfactory habituation/dishabituation tests, whereas females displayed no significant deficits in these tasks. Conversely, VPA‐exposed female rats exhibited anxiety‐like behaviours in the EPM and OFT, while males were unaffected in these paradigms. These results highlight a sexually dimorphic effect of prenatal VPA exposure on neurobehavioural outcomes, suggesting distinct vulnerability patterns in social and anxiety‐related domains between sexes.

Mitochondrial dysfunction is reported in ASD, with increased pyruvate and lactate levels in individuals with ASD being associated with decreased pyruvate dehydrogenase activity (Giulivi et al., 2010). Altered lactate levels may contribute to elevated blood glucose levels in ASD, as lactate is metabolized to glucose in the liver (Manco et al., 2021). In healthy rats, no sex differences have been observed in blood glucose levels (Gustavsson et al., 2010). Kim et al. reported increased serum glucose levels in male rats that were exposed to 400 mg/kg of VPA In utero (H. Y. Kim et al., 2022). In another study, metformin treatment ameliorated ASD‐like symptoms and restored antioxidant activity in rats with prenatal VPA exposure (Ishola et al., 2020). Metformin is a commonly used pharmacological agent for diabetes. Our results are in line with the literature for VPA‐exposed males. VPA‐exposed females, on the other hand, displayed no differences compared to controls. Blood glucose levels are controlled by several homeostatic mechanisms. Leptin, for example, acts to decrease glucose levels (Fernández‐Formoso et al., 2015), whereas ghrelin has hyperglycaemic effects (Dezaki et al., 2004). The presence of decreased glucose levels in VPA‐exposed males, unlike VPA‐exposed females, may be attributed to the decreased leptin and increased ghrelin levels that we reported in VPA‐exposed males.

Orexins are important for sleep regulation and energy metabolism, and their levels increase with food intake. We measured orexin‐A levels, as it can cross the blood–brain barrier (Mediavilla, 2020). Kobylinska et al. (2019) reported increased plasma orexin‐A levels in children with ASD. However, this increase was negatively correlated with symptom severity. In patients with AN, decreased orexin‐A levels have been associated with heightened anxiety (Steward et al., 2019). While no sex differences in plasma orexin‐A levels have been reported in healthy subjects (Arihara et al., 2001), female rats have been shown to exhibit higher orexin‐A levels in the hypothalamus and cerebrospinal fluid (Grafe et al., 2017). Despite the fact that orexin‐A levels have not been studied in ASD models, the use of an orexin receptor antagonist has been shown to alleviate ASD‐like symptoms in VPA‐exposed rats (Piri et al., 2024). Although our results showed no differences in orexin‐A levels in serum, VPA females displayed higher levels in brain homogenates compared to both control females and VPA males. Control females also showed higher orexin‐A levels in the brain compared to control males. These results are in line with previous studies regarding orexin‐A's sex distribution. As mentioned above, lower levels of orexin‐A are associated with increased anxiety. In this sense, the increased entry to open arms observed in VPA females may be associated with higher orexin‐A levels.

Nesfatin‐1 is an anorexigenic hormone involved in glucose homeostasis, reducing food intake and increasing glucose uptake into tissues. Çelikkol Sadıç et al. (2021) reported no difference in plasma nesfatin‐1 levels between children with ASD and neurotypical children. Serum nesfatin‐1 levels are reported to be higher in healthy male rats compared to females (Catak et al., 2014). Increased nesfatin‐1 levels have been associated with elevated anxiety. Bez et al. (2012) reported increased plasma nesfatin‐1 levels in patients with obsessive–compulsive disorder. Merali et al. (2008) showed increased anxiety and fear conditioning in rats after i.c.v. application of nesfatin‐1. To our knowledge, there is no study investigating nesfatin‐1 levels in ASD models. We observed an increase in serum and a decrease in brain tissue in VPA‐exposed animals.

Leptin is a hormone involved in energy metabolism and puberty. It has been reported that plasma leptin levels were not different in children with ASD (Skórzyńska‐Dziduszko et al., 2023). However, Al‐Zaid et al. (2014) reported higher plasma leptin levels in boys with ASD. Serum leptin levels are reported to be higher in female rats before puberty (Connor et al., 2012) but lower after puberty (Mulet et al., 2003). In the VPA model of ASD, leptin treatment has been reported to improve ASD‐like behaviours and reduce inflammation (Hamzawy et al., 2018). Our results showed decreased serum leptin levels in VPA‐exposed animals, consistent with Hamzawy et al.'s findings. Decreased serum leptin and increased blood glucose levels observed in VPA‐exposed males are consistent with leptin's role in glucose homeostasis (Boucsein et al., 2021).

Ghrelin is a stomach‐derived hormone that acts as a link between metabolism and cognition. Its roles include stimulating growth hormone release (Asakawa et al., 2001), synaptogenesis in the hippocampus (Bourgeron, 2009), promoting food intake, and mediating stress (Chen et al., 2022). Bagheri et al. (2015) reported no difference in ghrelin levels in pre‐pubertal children, while Soriano‐Guillen et al. (2016) reported higher ghrelin levels in girls during puberty. Similar results have been observed in rodents, with females having higher ghrelin levels, which are influenced by oestradiol (Börchers et al., 2022). Recent studies suggest a possible role for ghrelin in ASD. As mentioned, ghrelin plays a role in synaptogenesis, and abnormal synaptogenesis has been reported in ASD (Bourgeron, 2009). Leptin and ghrelin have a reverse relationship, as leptin suppresses ghrelin transcription (Li & Ford, 1998). While there are contradictory results regarding leptin levels in ASD, Al‐Zaid et al. (2014) reported higher leptin levels with lower ghrelin levels in boys with ASD. Our results showed a reversed interaction between these two molecules, consistent with their known relationship pattern. We observed higher serum ghrelin and lower serum leptin levels in the VPA groups, differing from individuals with ASD. However, a circadian rhythm study of ghrelin and leptin in rats showed opposite levels in the light compared to humans (Keleş et al., 2018). This study reported that 12‐h food‐deprived rats had higher leptin and lower ghrelin levels during the light phase compared to the dark phase (Bodosi et al., 2004). Çelikkol Sadıç et al. (2021), however, reported higher ghrelin levels in toddlers with ASD. In our study, increased serum ghrelin levels and decreased locomotor activity in the VPA group support the reported negative effect of ghrelin on locomotion (Pfluger et al., 2008).

The current study has a relatively small sample size used for behavioural and molecular testing in VPA‐exposed rats. While this may affect the generalizability of the findings, the results still provide valuable preliminary insights into the alterations of metabolism‐related molecules following VPA exposure. It should be noted that using two pups per litter presents a limitation in terms of litter effect. Consideration of different analyses methods, such as making litter a random factor in a linear effects model, would be beneficial to the study design (Golub & Sobin, 2020; Lazic & Essioux, 2013). Despite these limitations, the presented data contribute to the growing body of research on VPA‐induced behavioural and metabolic phenotypes and may serve as a foundation for further investigations.

In conclusion, this study utilized In utero VPA exposure to model ASD‐like behaviours in both female and male rats. The validity of the model was confirmed by testing communication deficits through olfactory discrimination and social interaction problems using the three‐chambered social performance test. Additionally, locomotor activity, anxiety and exploratory behaviour were assessed, revealing alterations due to VPA exposure. The results are consistent with previous studies.

Metabolic and eating‐related disruptions are commonly reported in individuals with ASD. However, research into the interaction of key peptides involved in eating regulation and ASD remains limited, to the best of our knowledge. Our findings demonstrate altered brain and serum levels of orexin‐A, leptin, nesfatin‐1 and ghrelin in the prenatal VPA‐exposed rat model of ASD, with sex considered as a biological variable.

Females with ASD are often underdiagnosed or misdiagnosed with anxiety, depression, or eating disorders. Addressing the male bias in ASD research requires not only more inclusive human studies but also the strategic use of models like In utero VPA exposure. Including females in ASD research, despite the male biases, is essential for achieving a more equitable understanding of autism and improving diagnosis, treatment and support for all individuals on the spectrum, regardless of sex.

## AUTHOR CONTRIBUTIONS

Süeda Tunçak contributed to study design, carrying out experimental processes, data collection, data analyses, and initial draft of paper. Ayşen Çakır contributed to study design, funding acquisition, carrying out experimental processes, data collection, data analyses, and initial draft of paper. Bülent Gören contributed to study design, provided critical reviews on analyses and initial draft of the paper. Nevzat Kahveci contributed to study design, provided critical reviews on analyses and initial draft of the paper. All authors have read and approved the final version of this manuscript and agree to be accountable for all aspects of the work in ensuring that questions related to the accuracy or integrity of any part of the work are appropriately investigated and resolved. All persons designated as authors qualify for authorship, and all those who qualify for authorship are listed.

During the preparation of this work, the authors used ChatGPT in order to language check. After using this tool, the authors reviewed and edited the content as needed and take full responsibility for the content of the publication.

## CONFLICT OF INTEREST

The authors declare no conflicts of interest.

## Data Availability

The data that support the findings of this study are available from the corresponding author upon reasonable request.
